# Stridulations Reveal Cryptic Speciation in Neotropical Sympatric Ants

**DOI:** 10.1371/journal.pone.0015363

**Published:** 2010-12-22

**Authors:** Ronara Souza Ferreira, Chantal Poteaux, Jacques Hubert Charles Delabie, Dominique Fresneau, Fanny Rybak

**Affiliations:** 1 Laboratoire d'Ethologie Expérimentale et Comparée, LEEC EA 4443, Université Paris 13, Villetaneuse, France; 2 UPA Laboratorio de Mirmecologia, Convênio UESC/CEPLAC, Centro de Pesquisas do Cacau, Itabuna, Brazil; 3 Univ Paris-Sud, Centre de Neurosciences Paris-Sud, UMR 8195, Orsay, France; 4 CNRS, Orsay, France; University of Saint-Etienne, France

## Abstract

The taxonomic challenge posed by cryptic species underlines the importance of using multiple criteria in species delimitation. In the current paper we tested the use of acoustic analysis as a tool to assess the real diversity in a cryptic species complex of Neotropical ants. In order to understand the potential of acoustics and to improve consistency in the conclusions by comparing different approaches, phylogenetic relationships of all the morphs considered were assessed by the analysis of a fragment of the mitochondrial DNA cytochrome b. We observed that each of the cryptic morph studied presents a morphologically distinct stridulatory organ and that all sympatric morphs produce distinctive stridulations. This is the first evidence of such a degree of specialization in the acoustic organ and signals in ants, which suggests that stridulations may be among the cues used by these ants during inter-specific interactions. Mitochondrial DNA variation corroborated the acoustic differences observed, confirming acoustics as a helpful tool to determine cryptic species in this group of ants, and possibly in stridulating ants in general. Congruent morphological, acoustic and genetic results constitute sufficient evidence to propose each morph studied here as a valid new species, suggesting that *P. apicalis* is a complex of at least 6 to 9 species, even if they present different levels of divergence. Finally, our results highlight that ant stridulations may be much more informative than hitherto thought, as much for ant communication as for integrative taxonomists.

## Introduction

The Tropics are home to nearly two-thirds of the World's known biodiversity, but also to a large amount of species that have remained unnoticed [1], partly due to the occurrence of cryptic species, i.e. of two or more distinct species that are erroneously classified (and hidden) under a single species name, due to their very similar morphology [2]. Such species cause great problems for taxonomists as they cannot be readily or reliably distinguished only on a morphological basis, and the taxonomic challenge they pose underlines the importance of using multiple criteria in species delimitation. Indeed, concordant changes in several characteristics of an organism, and corroboration from independent data constitute better evidence for separating species [2], [3]. As accurate species identification is crucial both to research in all areas of biology and to biodiversity conservation [2], [4], there is an urgent need to coalesce effective tools which allow the clarification of these taxonomic issues in order to estimate the real biodiversity in cryptic species groups.

Acoustic signals may differ between cryptic species and were often the first clue of a hidden diversity in many insect groups, like hemipterans [5], lacewings [6], orthopterans [7]–[9] and flies [10], [11]. By delimitating species phenotypic variability, acoustic analyses have revealed many unsuspected species and solved several confusing taxonomic problems. Thus, acoustic descriptions should be systematically included in species diagnosis [12]. The use of DNA sequences (“DNA barcoding”) in extensive phylogenetic studies has also revealed an effectively high level of hidden biodiversity [13]–[16], suggesting that molecular data should be incorporated by taxonomists as a matter of routine [2].

In ants, stridulatory sound production is known since the late nineteenth century [17]–[21]. This faculty seems to have evolved several times independently and can be found in a great number of species from five subfamilies (Myrmicinae, Pseudomyrmecinae, Ponerinae, Ectatomminae, Nothomyrmecinae) [22], [23]. Stridulations are produced during dorso-ventral movements of the gaster by the rubbing of the distal border of the 3rd segment of the abdomen that acts like a scraper on the *stridulatory file*, made up of perfectly parallel and rectilinear tegument ridges on the mid-dorsal edge of the fourth abdominal segment [24], [25]. They consist in *chirps* made by train of pulses, in which each pulse corresponds to the rubbing movement of the scraper on one ridge on the stridulatory file [26]. These signals are essentially transmitted by the substrate [27], [28], but evidence for a perception of air-transmitted sounds, at least over distances of a few centimetres, is also available [29]. These signals are generally barely audible without amplification [22], [29] but in some Ponerinae species, like *Pachycondyla apicalis*, the intensity of the airborne sound emitted by a single ant at a distance of 1 cm can reach more than 93 dB [30]. The frequency of the signal can vary from a few hertz, like in *Solenopsis richteri* [29] and *Myrmica spp* [31], [32] to up to 84 kHz in *P. apicalis* [30]. Thus far, stridulations have been shown to be produced in several behavioural contexts depending on the species examined, like food recruitment [27], [33]–[34], trophallaxis [35], nest emigration [36], intra- and inter-specific conflicts [37], [38], and mating [39], [40]. Some ants can also respond to stridulations produced by their mutualists [41]–[44] or even their parasites [31]–[32].

However, even if stridulations are common events in ant societies, it is still probably the least understood mode of communication and detailed studies on the acoustic characteristics of these signals are scarce [45]. Furthermore, up to now, most work on ant stridulations refers only to the subfamily Myrmicinae, and almost nothing is known about primitive ants like the Ponerinae. In this ant subfamily, the genus *Pachycondyla* is one of the most ancient still living [26], [46]. It presents over 60 described species just for the Neotropics [47], and after some assessment studies [48]–[50], it appears to be a good bioindicator of the myrmecological diversity and quality of Neotropical ecosystems. Recent studies [51]–[53] demonstrated that many Neotropical *Pachycondyla* actually consist of cryptic species complexes, indicating that the diversity in this genus can be really underestimated.

The *Pachycondyla apicalis* species complex is a good example of this problem and its taxonomy remains unsettled. Ants in this group are large, conspicuous insects found in Neotropical forests from southern Mexico to Paraguay, overall presenting similar ecological and biological features [54], [55]. Furthermore, ants in this complex also share the same general morphology and coloration (the “morph *apicalis*”) which is probably under stabilizing selection as it is also copied by other arthropods like spiders [56]–[58]. These sympatric species could thus form a massive mimicry ring which would represent an advantage against predators [15], [57], [59]. Wild [54] revised the taxonomy of this group using several morphological and biometrical criteria and recognised three broadly sympatric species: *P. apicalis* [60], *P. obscuricornis* [61] and *P. verenae* [62] instead of only two as thought before [63]. Although he observed a considerable morphological variation for *P. verenae* and *P. apicalis* across their distribution ranges he did not consider further division of the complex. Later, in a morphological study combining cytogenetical and ecological data when available, Delabie et al. [59] demonstrated that, given the stability of differences through multiple cases of sympatry, the variability pointed by Wild [54] could in fact refer to a species mosaic rather than a geographic cline. They recognized seven distinct taxa within this group: four for *P. apicalis*, two for *P. verenae* and one for *P. obscuricornis*, and despite supporting a really intricate diversity inside the *P. apicalis* species complex, the authors were not able to conclude about the validity and the taxonomic status (e.g. species, subspecies, ecotypes) of the different morphs within each species without further investigations.

Here for the first time, we test acoustics as a tool to assess the real diversity in a cryptic species complex of Neotropical ants. We measured the overall structure of the stridulatory file and performed a detailed acoustic analysis of the stridulations produced in five morphs of the *P. apicalis* species complex. To improve the consistency in conclusions by comparing different approaches, we also analysed a fragment of the mitochondrial DNA cytochrome b for all morphs considered.

## Methods

### Ants

Colonies of the *P. apicalis* species complex were collected at Petit Saut, French Guiana (n = 10) and Los Tuxlas, Mexico (n = 1). Colonies were reared in the laboratory in artificial plastered nests. The nests were maintained at 25±1°C, with approximately 65±10% relative humidity, and a 12L∶12D photoperiod. All colonies were provided with an identical diet (honey/apple mixture and crickets) twice a week. Ant collection, husbandry and experimental procedures used in this study fulfilled all the legal requirements concerning insect experimentation of France.

Ants were classified into morphs within the currently named species according to Delabie et al. [59] classification. Three different morphs could be identified, one for *P. verenae* (PVE) and two for *P. apicalis* (PAP), respectively: PVE Morph 1 (5 colonies), PAP Morph 3 (1 colony) and PAP Morph 4 (2 colonies) (see supplementary Figures S1, S2 and S3). Among our collected colonies, we did not find the *P. verenae* Morph 2 or *P. apicalis* Morphs 1 or 2. Moreover, three colonies of *P. apicalis* from Petit Saut did not fit any described morph in Delabie et al. [59]. We treated them here as 2 new morphs: PAP Morph 5 (2 colonies) and PAP Morph 6 (1 colony), as they also presented subtle distinctive morphological traits. PAP Morph 5 is moderate sized, with an emarginated petiole and a very finely striated cuticle and hairy head and body (see supplementary Figure S4). PAP Morph 6 presents a rounder petiole and is clearly bigger than all other *P. apicalis* examined (see supplementary Figure S5). PVE Morph 1 and PAP Morphs 4, 5, and 6 were found in sympatry in the same site at Petit Saut, French Guyana. PAP Morph 3 is allopatric to all other morphs and occurs only in Mexico [59]. Vouchers of each morph were deposited in the CPDC collection of the Laboratório de Mirmecologia, Cocoa Research Center at Itabuna (Bahia, Brazil).

### Morphometric Study of the Stridulatory Organ

**Figure 1:**
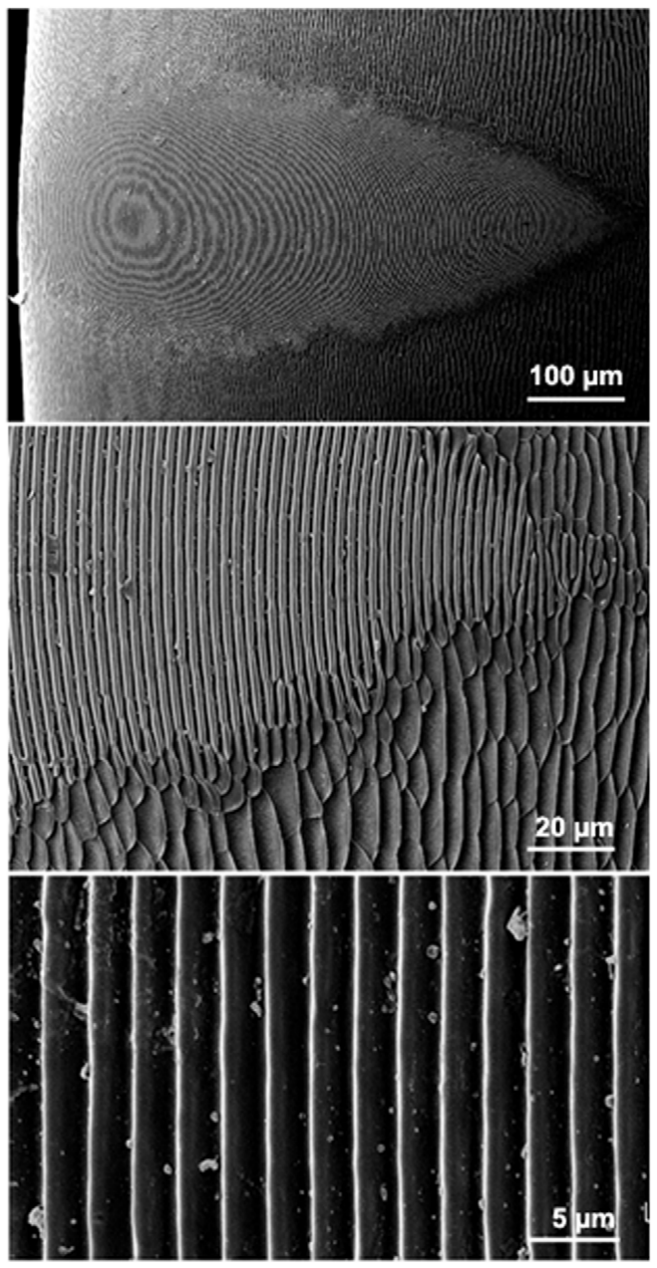
Scanning electron micrographs of the stridulatory file of a *Pachycondyla apicalis* species complex worker. (A) General view of the stridulatory file, (B) detail of the ridges showing the interface between the fine structure of the file and the surrounding cuticle and (C) detail in the medial portion of the stridulatory file, showing the inter-ridge distances.

A total of 41 workers from 8 colonies (Table 1) were dissected and the segments of the gaster containing the stridulatory file were cleaned in an ultrasonic-wave bath, air-dried and then placed on aluminium stubs. The samples were coated with a mixture of 80% gold/20% palladium and examined with a Leica Stereoscan 440 scanning electron microscope (SEM). Measurements of the stridulatory organ were obtained from the digitalized SEM images. Eight variables were measured from each stridulatory file: Length, maximal width, 1^st^, 2^nd^ and 3^rd^ quartile widths, number of ridges and inter-ridge distance in the medial and distal portions of the stridulatory file (Figure 1). For these two last variables, 5 measures were taken from each worker and the mean value was computed. Also, as an estimate of the ants' size, the thorax length of 42 other workers was measured with a Zeiss Stereo Microscope at a magnification of 10x.

**Table 1:** Morphometric characteristics (mean±SE) of the stridulatory file in five morphs of the *Pachycondyla apicalis* species complex.

FILE OF RIDGES	*P. verenae* Morph 1	*P. apicalis* Morph 3	*P. apicalis* Morph 4	*P. apicalis* Morph 5	*P. apicalis* Morph 6
Number of ridges	245.10±2.86	212.71±4.76	242.78±5.03	242.60±4.32	258.40±8.97
Length (µm)	574.22±8.41	618.88±10.63	624.43±16.84	617.38±8.06	693.72±18.42
Maximal width (µm)	282.12±7.43	234.63±5.14	239.60±4.71	279.24±3.90	214.48±4.14
1st quartile width (µm)	272.46±6.67	209.09±7.42	232.80±4.64	260.86±6.46	214.48±4.14
2nd quartile width (µm)	216.73±10.34	232.33±6.22	177.84±6.85	230.91±5.08	143.64±3.75
3rd quartile width (µm)	161.04±5.63	143.19±9.03	135.63±4.82	146.23±3.23	73.44±5.14
Mean inter-ridge distance median region (µm)	2.51±0.04	2.89±0.03	2.86±0.07	2.58±0.06	3.07±0.08
Mean inter-ridge distance distal region (µm)	2.35±0.04	3.02±0.03	2.55±0.07	2.81±0.06	2.80±0.08
# Workers (# Colonies)	10 (2)	7 (1)	9 (2)	10 (2)	5 (1)

### Stridulation recording and analysis

**Figure 2:**
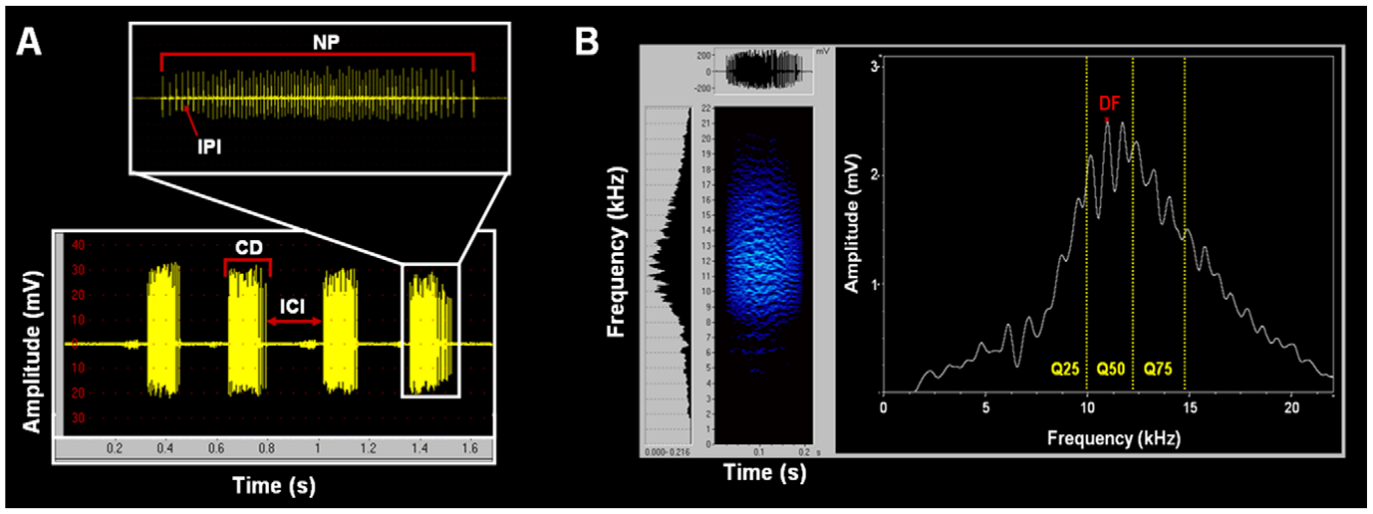
Stridulatory signal of a *Pachycondyla apicalis* species complex worker. (A) Oscillogram of a series of chirps, showing the chirp duration (CD), the inter-chirp interval (ICI), the number of pulses (NP) in a chirp and the inter-pulse interval (IPI). (B) Spectrogram of a chirp, showing the dominant frequency (DM) and the quartiles of frequencies at 25, 50 and 75% of the signal energy (Q25, Q50 and Q75 respectively).

Ants from the *P. apicalis* species complex produce stridulations which result in both airborne sound and substrate vibrations. Airborne sound presents audible and ultrasound components, and in the following we refer to the audible component only. A total of 40 workers from 5 colonies (Table 2) were recorded. All recordings were carried out in a low-noise room where the ambient temperature was kept at 25±1°C and the relative humidity at 65±10%. The recording setup consisted of an omnidirectional Sennheiser K6 microphone (frequency response: 30 to 20000 Hz±1 dB) connected to a Marantz PMD 671 digital recorder with sampling frequency at 48 kHz. We did not consider frequencies superior to 20 KHz, due to technical limitations of the microphone. Ants were held with forceps 1 cm from the microphone during recording. The following *temporal parameters* were analysed using the software Avisoft-SASLab Pro, version 4.40 [64]: the chirp duration, the inter-chirp interval, and for each chirp we measured the number of pulses, the pulse repetition rate, the mean inter-pulse interval as well as the inter-pulse interval in the 1^st^, 2^nd^ and 3^rd^ thirds of the chirp. The *frequency parameters* considered for each chirp were: the dominant frequency, the frequencies at 25, 50 and 75% of the signal energy, and the percentage of energy below 14 kHz. The maximal and minimal intra-pulse frequencies were also calculated for each individual, by the zero-crossing method [65]. For each chirp analysed, we calculated the maximal and minimal intra-pulse frequencies for 10 pulses and the mean value was computed (Figure 2). A series of ten chirps was analyzed for each ant, and the mean value was computed.

**Table 2:** Acoustic characteristics (mean±SE) of the stridulatory signals in five morphs of the *Pachycondyla apicalis* species complex.

STRIDULATIONS	*P. verenae* Morph 1	*P. apicalis* Morph 3	*P. apicalis* Morph 4	*P. apicalis* Morph 5	*P. apicalis* Morph 6
Chirp duration (ms)	105.83±3.67	141.25±7.97	162.87±8.76	128.89±7.07	150.17±13.18
Inter-chirp interval (ms)	286.82±40.94	245.46±20.80	225.59±9.49	229.86±9.53	436.65±77.02
Number of pulses	77.68±7.83	95.70±7.90	96.78±5.28	98.69±8.08	121.59±6.64
Pulse repetition rate (Hz)	695.85±79.01	682.70±85.83	605.17±37.15	771.81±62.89	856.87±106.73
Mean Inter-pulse interval (ms)	1.38±0.17	1.55±0.22	1.61±0.12	1.26±0.11	1.20±0.16
Inter-pulse interval 1st third (ms)	1.24±0.16	1.52±0.18	1.39±0.15	1.14±0.10	1.12±0.10
Inter-pulse interval 2nd third (ms)	1.05±0.14	1.18±0.16	1.25±0.10	0.96±0.09	0.98±0.11
Inter-pulse interval 3rd third (ms)	1.84±0.23	1.95±0.33	2.18±0.13	1.67±0.14	1.50±0.29
Dominant frequency (kHz)	10.56±0.65	11.10±0.27	12.26±0.41	10.77±0.25	11.85±0.15
Frequency at 25% energy (kHz)	8.90±0.14	9.31±0.11	9.70±0.25	9.18±0.11	9.40±0.15
Frequency at 50% energy (kHz)	11.40±0.21	11.79±0.08	12.24±0.20	11.72±0.13	12.06±0.12
Frequency at 75% energy (kHz)	14.55±0.17	14.71±0.07	14.64±0.13	14.67±0.13	14.87±0.08
Proportion of energy below 14 Khz	0.71±0.01	0.69±0.01	0.68±0.01	0.70±0.01	0.68±0.01
Minimal intra-pulse frequency (kHz)	9.13±0.07	9.12±0.20	11.53±0.43	9.31±0.22	9.47±0.41
Maximal intra-pulse frequency (kHz)	10.82±0.33	11.54±0.12	12.22±0.27	11.57±0.12	11.93±0.17
# Workers	6	8	9	9	8

### DNA extraction, amplification and sequencing

The DNA of 22 workers from 11 colonies was extracted from ethanol-preserved tissues (head and thorax) using a DNeasy Blood & Tissue kit (Qiagen Inc., Valencia, CA) following the manufacturer's protocol. Mitochondrial DNA variation was assayed by the amplification of a portion of the mtDNA cytochrome b (cyt b, ∼700 bp) using primers CB1 (5′-TAT-GTA-CTA-CCA-TGA-GGA-CAA-ATA-TC-3′) and tRS (5′-TAT-TTC-TTT-ATT-ATG-TTT-TCA-AAA-C-3′) from Simon et al. [66]. Each PCR was carried out in a 50-µL volume according to a standard protocol using a T1 thermal cycler (Biometra). The thermal cycle profile was as follows: 2 min at 94°C; 35 cycles at [30 s at 94°C/60 s at 50°C/60 s at 72°C]; 5 min at 72°C. Amplified products were sequenced using the same primers as used for the amplification by Genoscreen (Lille, France) using an ABI 3730XL automatic sequencer (Applied Biosystems).

Sequences analyses were edited and aligned using the default settings of Clustal X [67] and checked by eye. To generate phylogenetic trees, we used pairwise distances (Neighbor Joining algorithm, NJ). As *Pachycondyla* is a paraphyletic genus (C. Schmidt, pers. com.), we used two other divergent *Pachycondyla* species as outgroups: *P. villosa* and *P. goeldi*, respectively. Average intermorph genetic divergence was calculated using the Kimura 2-parameter model [68] using the MEGA4 program [69]. Other models (Jukes–Cantor or Tamura-Nei distance) when applied, resulted in similar results. NJ tree was constructed with the MEGA4 program. The robustness of the tree was tested with 1000 bootstrap replications. The equality of evolutionary rate between all sequences was tested using Tajima' relative rate test [70] in MEGA4.

### Statistical analysis

Workers used for the morphometric measurements came from two different colonies for PVE Morph 1 and PAP Morphs 4 and 5. As these colonies did not differ for the structure of the stridulatory organ (MANOVA “colonies nested within morphs”, Wilk's λ = 0.249, F_24,47_ = 1.205, p = 0,2863), their data were pooled for further comparisons between morphs. Discriminant function analyses (DFA) were performed to identify potential differences of the stridulatory file morphometry and the acoustic characteristics of stridulations between morphs and to calculate the success rate of individual discrimination (correct classification rate) using Mahalanobis distances. As the temporal parameter of stridulations “inter-chirp interval” showed a very high coefficient of variation within morphs (ranging from 13.31 to 45.50%), this parameter was not included in the DFA. Additionally, we compared each morphometric and acoustic parameter using one-way ANOVA followed by Unequal N HSD (Honest Significance Difference) post hoc tests [71] to understand how these parameters varied between morphs. Paired Student's t-tests were used to establish the differences between the medial and distal inter-ridge distances within groups. When necessary, Box-Cox transformation was applied to achieve normality on some parameters [72]. All results are stated as mean±SE. The significance level was taken at α≤0.05 to assess differences. All analyses were conducted using Statistica v8.0 [73].

## Results

### Morphometry of the stridulatory organ

**Figure 3:**
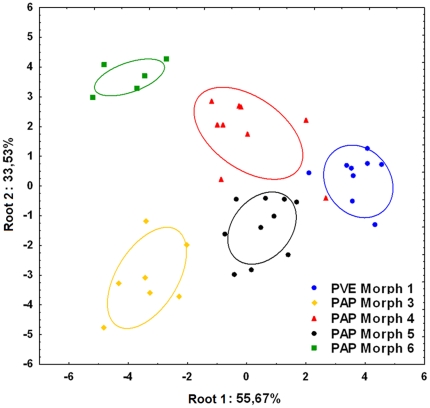
Discriminant function analysis of the stridulatory file morphometry of the *P. apicalis* species complex. PVE: *P. verenae*, PAP: *P. apicalis.* Ellipses are 95% confidence intervals around centroids.

**Figure 4:**
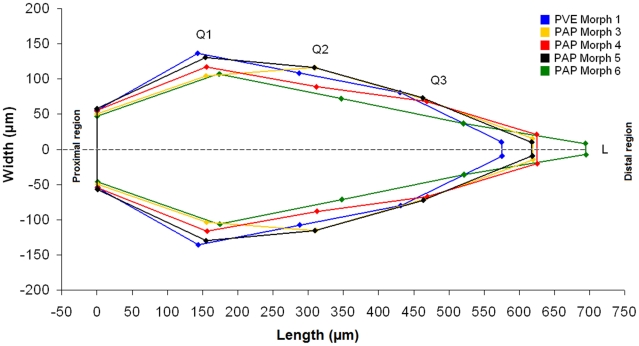
Pattern of the stridulatory file of five morphs from the *P. apicalis* species complex. First (Q1), second (Q2) and third (Q3) quartile widths of the file of ridges in relation with the length (L). ANOVA_Q1_, F_4,36_ = 18.74, ANOVA_Q2_, F_4,36_ = 20.25, ANOVA_Q3_, F_4,36_ = 25.88 and ANOVA_Length_, F_4,36_ = 9.82. All p<0.001. PVE: *P. verenae*, PAP: *P. apicalis*.

**Figure 5:**
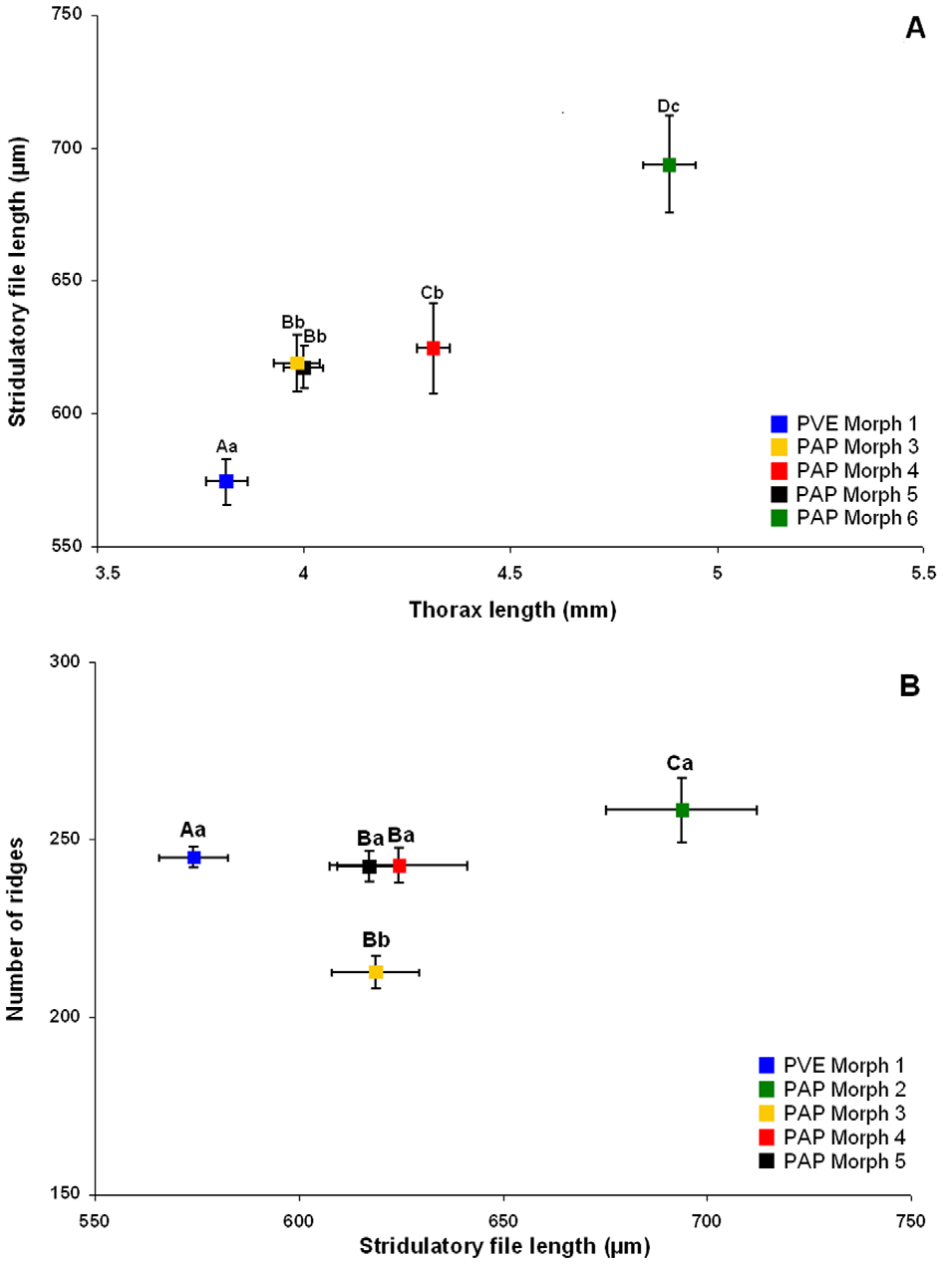
Ant size and stridulatory file features. Relationship between the stridulatory file length and (A) the ant size (represented by thorax length) and (B) the number of rigdes of five morphs from the *P. apicalis* species complex. (ANOVA_Thorax_, F_4,37_ = 57.82, p<0.001, ANOVA_File_, F_4,36_ = 9.83, p<0.001 and ANOVA_Ridges_, F_4,36_ = 9.66, p<0.001). When different, capital letters indicate significant differences for parameters on the X axes, and small letters indicate significant differences b for parameters on the Y axes tests, p<0.05. PVE: *P. verenae*, PAP: *P. apicalis*.

**Figure 6:**
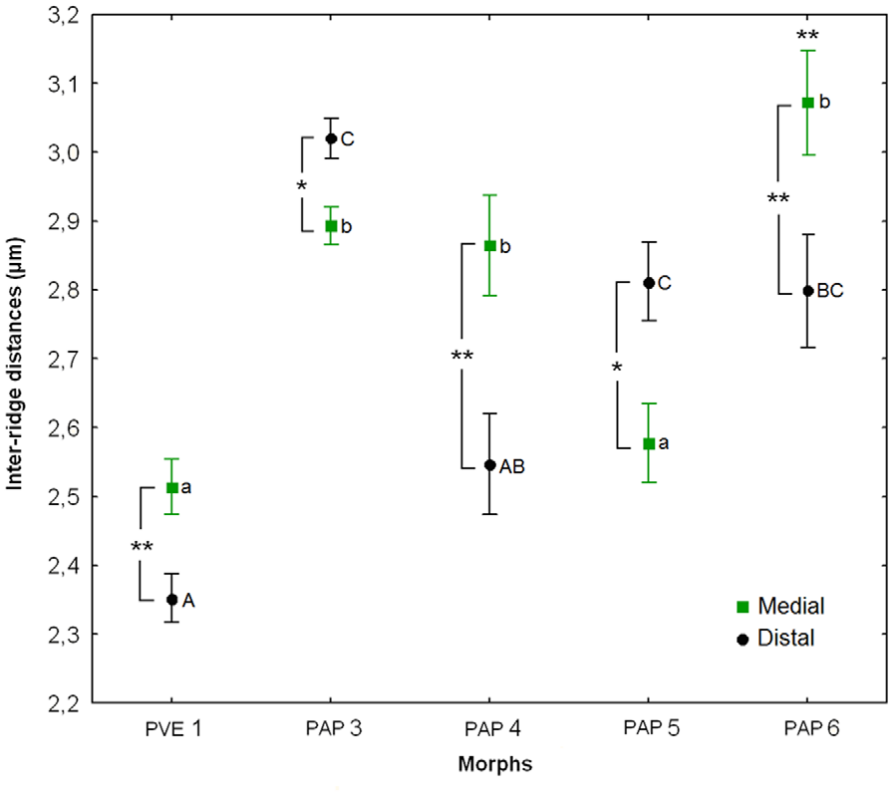
Inter-ridge distances of the stridulatory file in the *P. apicalis* species complex. Comparison of the inter-ridge distances in the medial and distal portions of the stridulatory file within morphs (Paired Student's t-tests within morphs, t_PVE Morph 1_ = 4.28, t_PAP Morph 3_ = −3.11, t_PAP Morph 4_ = 3.86, t_PAP Morph 5_ = −2.93, t_PAP Morph 6_ = 7.18; * p<0.05 and ** p<0.01) and between morphs (ANOVA_Medial_, F_4,36_ = 15.17, p<0.001, ANOVA_Distal_, F_4,36_ = 21.61, p<0.001. When different, small letters indicate significant differences of inter-ridge distances on the medial portion of the stridulatory file and capital letters indicate significant differences of inter-ridge distances on the distal portion of the stridulatory file (Unequal N HSD tests, p<0.05)). PVE: *P. verenae*, PAP: *P. apicalis*.

The discriminant function analysis of all morphometric parameters considered for the stridulatory file clearly separates all studied morphs (Wilk's λ = 0.005, F_32,108_ = 10.712, p<0.001; Figure 3, Table 1). The file (see supplementary Figure S6) can vary in length (Figures 4 and 5.a), maximal and quartile widths (Figure 4), medial and distal inter-ridge distances (Figure 6) and number of ridges between morphs (Figure 5.b). These differences are combined in a distinctive way within each morph, which allows a 97.5% correct classification rate of all individuals based on the overall pattern of the stridulatory file.

Differences observed in the pattern of the stridulatory file are not due only to allometric differences between individuals. Morphs with different body size present the same length for the stridulatory file (Figure 5.a). Moreover, stridulatory files of the same length can comprise different number of ridges (Figure 5.b), which directly affect the inter-ridges distance. This latter parameter presents complex variation patterns along the stridulatory file depending on the morph. It can vary between the medial (ANOVA, F_4,36_ = 15.17, p<0.001) and distal (ANOVA, F_4,36_ = 21.61, p<0.001) regions of the file and the pattern is not the same for all morphs: PVE Morph 1 and PAP Morphs 4 and 6 present ridges more spaced in the middle than in the distal region of the file, while PAP Morph 3 and 5 present the opposite pattern (Figure 6).

### Acoustic analyses of stridulations

**Figure 7:**
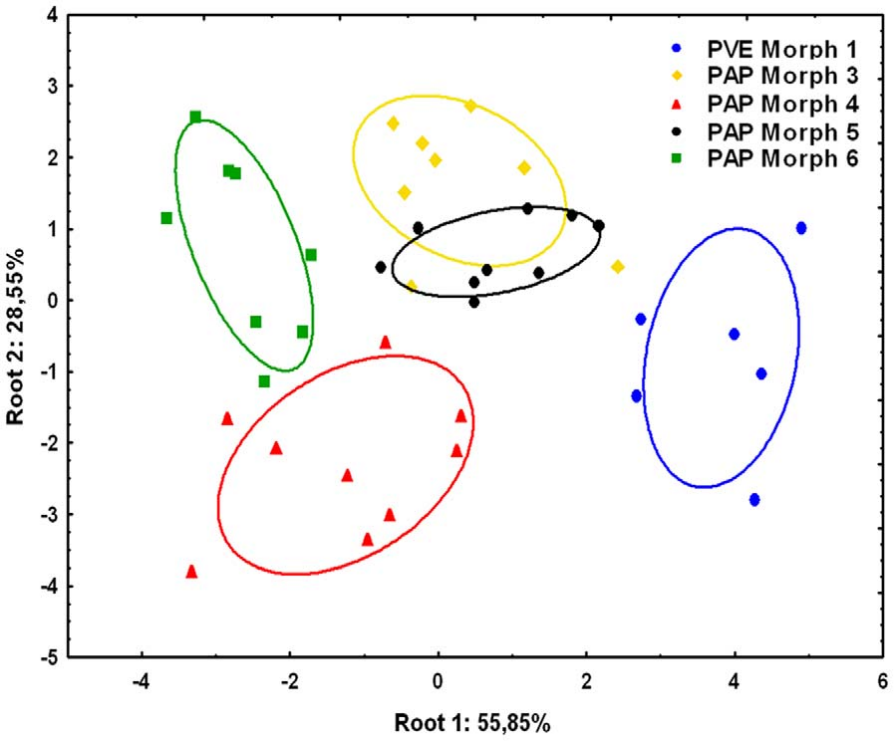
Discriminant function analysis of the stridulations of the *P. apicalis* species complex. All temporal and frequency parameters measured (except the inter-chirp interval) are considered in the model. PVE: *P. verenae*, PAP: *P. apicalis*. Ellipses are 95% confidence intervals around centroids.

The DFA of all temporal and frequency parameters (except the inter-chirp interval) reveals that each sympatric morph from French Guiana produce distinctive sounds (Wilk's λ: 0.01987, F_56,87_: 2.724, p<0,0001; Figure 7, see supplementary Audio S1, Audio S2, Audio S3, Audio S4 and Audio S5). Here, the differences in the acoustic characteristics of stridulations allowed a correct classification rate of 95% of all individuals. PVE Morph 1 produces shorter chirps than PAP Morphs 4 and 6 (ANOVA_Chirps_, F_4,35_ = 5.24, p<0.01, Unequal N HSD, p<0.01 and p<0.05 respectively, Table 2), and with fewer pulses than PAP Morph 6 (ANOVA_NPulses_, F_4,35_ = 4.18, p<0.01, Unequal N HSD, p<0.01, Table 2), notwithstanding the similarity in the number of ridges in the stridulatory file (Figure 5.b). This result is due to the smaller inter-ridge distances presented by PVE Morph 1 (Figure 6), but also to the smaller number of ridges that are rubbed during stridulatory movements (Table 2). In spite of presenting a high inter-individual variation, the inter-chirp interval in PAP Morph 6 is bigger than all the other PAP morphs (ANOVA_ICI_, F_4,35_ = 4.84, p<0.01, Unequal N HSD, p<0.05 for PAP Morph 3 and p<0.01 for PAP Morphs 4 and 5, Table 2), which means this morph produces fewer stridulations in a given time. Nevertheless, this morph seems to rub the stridulatory file faster during stridulation production, as suggested by the tendencies for a higher pulse repetition rate and a lower mean inter-pulse interval together with larger inter-ridge distances (Figure 6, Tables 1 and 2).

Concerning frequency parameters, PAP Morph 4 stridulations have higher dominant frequency than PVE Morph 1 and PAP Morph 5 (ANOVA_DF_, F_4,35_ = 4.21, p<0.01, Unequal N HSD, p<0.05 for both morphs, Table 2), higher frequencies at 25% and 50% of the signal energy than PVE Morph 1 (ANOVA_F25%_, F_4,35_ = 2.82, p<0.05, Unequal N HSD, p<0.05 and ANOVA_F50%_, F_4,35_ = 4.05, p<0.01, Unequal N HSD, p<0.05, Table 2), and higher intra-pulse minimal frequency than all other morphs (ANOVA_MFP_, F_4,35_ = 8.17, p<0.01, Unequal N HSD, p<0.01 for all morphs). The intra-pulse maximal frequency is also significantly lower in PVE Morph 1 compared to PAP Morphs 4 and 6 (ANOVA_LFP_, F_4,35_ = 5.62, p<0.01, Unequal N HSD, p<0.01 for PAP Morph 4 and p<0.05 for PAP Morph 6). Last, the stridulations of the allopatric PAP Morph 3 from Mexico did not significantly differ only from those of PAP Morph 5 from French Guiana (Figure 7) even if these morphs present distinctive stridulatory organs (Figures 3, 4, 5, 6).

### Nucleotide composition and sequence variation

**Figure 8:**
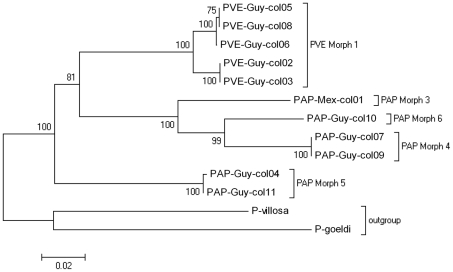
Neighbour-joining phylogenetic tree of mtDNA haplotypes of the *Pachycondyla apicalis* species complex. The tree is rooted using *P. villosa* and *P. goeldi* sequences as outgroups 1 and 2, respectively. Numbers indicate bootsrap values. PVE: *P. verenae*, PAP: *P. apicalis*.

A total of 690 base pairs were analysed for cyt b. Accession numbers range from HM770106 to HM770124 in Genbank. No pseudogenes, no insertions, deletions nor any rearrangements were detected. As in other hymenopteran mitochondrial genome [74], there is an A-T bias in the base composition of cyt b: we obtained on average 33.9% of A, 44.1% of T, 14.7% of C, and 7.3% of G. Over the 690 base pairs analysed, the number of variable characters was 259 among which 156 were found to be parsimony-informative. The transition/transversion rate ratios are *k_1_* = 1.207 (purines) and *k_2_* = 2.974 (pyrimidines), with an overall transition/transversion bias of 0.584. NJ tree of haplotypes obtained for the different morphs is presented in Figure 8, rooted with *P. villosa* and *P. goeldi* as outgroups. Tajima's relative tests are significant (X^2^ = 4.25, p = 0.038 to X^2^ = 8, p = 0.005, df = 1) when morphs of the clade constituted by *P. apicalis* are compared to *P. verenae* or PAP Morph 5, rejecting as a consequence the molecular clock hypothesis for this set of sequences.

We could observe 3 main groups, in which PAP Morph 5 clearly differs from all other morphs (about 15%), and presents a basal position to the *P. apicalis* and *P. verenae* groups (Figure 8).

Within *P. verenae* Morph 1, we obtain two clades but the level of divergence is low (mean distance of 1.5±0.3%). Within the group *P. apicalis*, the genetic distance between Morphs 3, 4 and 6 is considerable. When compared to the other morphs of *P. apicalis*, PAP Morph 3 varies in 10% of the base pairs considered, which nearly corresponds of the level of divergence between *P. verenae* and the clade *P. apicalis*. Between the sympatric *P. apicalis* Morphs 4 and Morph 6 the genetic variation is about 7% (Figure 8).

## Discussion

Our results demonstrate that each studied cryptic morph of the *P. apicalis* complex presents a morphologically distinct stridulatory organ, and that all sympatric morphs make distinctive sounds. The differentiation observed for the stridulatory organs is not only due to allometric differences between individuals but also to intrinsic morph features. The distinct acoustic signals produced, in their turn, are the result of this morphological specificity together with ant behaviour for stridulation production. Indeed, inter-specific competitive interactions in sympatry may have led to divergent selection acting in contrasting directions between morphs. In contrast, the similarity observed between the acoustic signals produced by PAP Morphs 3 and 5 in spite of the distinctiveness of their stridulatory files can be due to the fact that the allopatric PAP Morph 3 is not subjected to the same selective pressure as the sympatric morphs.

To our knowledge, we provide the first record of such a degree of acoustic specialization in closely related ants, both at the level of the production organ and of the produced signal. For a long time, stridulations have been considered as a mere generalist alarm signal that has no selective advantage to be species-specific and that could rarely be found specific even at the genus level [22]. In the few groups of sympatric and/or related species studied to date [three sympatric species of *Pogonomyrmex* ssp [39], four sympatric *Messor* species [38], [75], four Neotropical *Ectatomma* [30] and five *Myrmica spp.* [31], [32] no prominent inter-specific differences could be observed in the structures of the stridulatory file and in the characteristics of the signal. The only specificity already demonstrated in ant stridulations was at the intra-specific level, between different castes: for major and minor workers in *Atta cephalotes* [76] and for gynes and males or workers in four sympatric *Messor* species [38]. Recently, Barbero et al [31], [32] demonstrated that acoustic signals carry information about the caste and the status of a colony member in *Myrmica schencki* and trigger distinct behavioural responses by workers as a function of the identity of the emitter.

In ants, the most important channel for communication involves chemical and, to a smaller degree, tactile cues [26], [77]. However, the clear differentiation and specificity in the stridulatory file and signals observed in this study in a group of species with considerable morphological stasis suggests that acoustic communication may have a more important role than generally thought during interspecific relationships in these ants. We can also expect that, in this group of cryptic ants, acoustic signals might modulate chemical and tactile cues in different ways [31], and that a synergy between distinct-source information might improve communication in different behavioural contexts.

The variations observed here for some acoustic parameters would have likely been decreased and the differences between morphs would have likely been built up by studying individuals coming from different colonies for each morph. Nevertheless, mitochondrial DNA variation estimated for cytochrome b well supports the acoustic differences observed for all five morphs and confirms the taxonomic potential of acoustics for this group of cryptic ants and possibly for other stridulating ant species. We thus found that each studied morph in the *P. apicalis* species presents a real genetic identity. The most surprising result is the basal position of PAP Morph 5 and its high level of genetic divergence compared to other sympatric *P. apicalis.* The genetic isolation of PAP Morph 5 is mirrored by some morphological characters that could have been neglected, leaving this morph hidden until now. Moreover, despite the non-divergence of PAP Morph 5 stridulations from those of the allopatric PAP Morph 3, the corroboration of the distinct morphometry of the stridulatory file, genetics and morphology are enough evidence to separate these two taxa. Similarly, the genetic distances observed between the other two morphs of *P. apicalis,* even if lower than between PAP Morphs 3 and 5, are all well above the usual inter-specific values found for some cryptic insects groups, i.e. the 3% sequence divergence threshold typically used in the barcoding studies [78].

The congruence of the genetic data with acoustic and morphological information leads us to propose each morph studied here as a valid new species. In fact, the subtle morphological variations observed for these ants confirm not to be random, and what Wild [54] previously thought to be only intra-specific morphological variation, is verified by acoustic analysis and genetics to be distinct inter-specific traits, as supposed by Delabie et al. [59]. Given that acoustics matched the mitochondrial DNA divergence in the *P. apicalis* species complex and showed a high potential for species diagnosis in this group of cryptic species, further studies should consider including this tool to investigate cryptic diversity in stridulating ants. Indeed, with all the specific acoustic and morphological traits evidenced by the present study, the cryptic species in the *P. apicalis* complex can be considered from now on as pseudo-cryptic species, i.e. cryptic species for which after detailed comparisons of morphological and non-morphological features key characters can be established for their identification [3], [79]. In addition, further work should try to compare the type specimens of the described species, seeking to verify which morphs are the true *P. apicalis* [60] and *P. verenae* [62], and even *P. flavicornis* [60], a related *P. apicalis* species described by Latreille at the same time as *P. apicalis*, but synonymised later by Brown [63].

Speciation is a very complex process which is affected by many different factors (genetic, ecological, developmental, environmental, etc.) interacting in nonlinear ways [80]. The integrative approach undertaken in this paper was thus essential for the recognition of the real biodiversity in our sample of morphs of the *P. apicalis* species complex. Yet, the biodiversity inside this complex could even be more important than shown here. In this study, we had access to only three of seven morphs described in Delabie et al. [59] (*P. verenae* Morph 1 and *P. apicalis* Morphs 3 and 4) and to two new sympatric morphs of *P. apicalis* that we identified for French Guiana (*P. apicalis* Morphs 5 and 6). When taking into consideration that within the morphs non studied here there are still the ‘rare’ *P. obscuricornis*, a second morph of *P. verenae* and two additional *P. apicalis* morphs [59], the diversity inside the *P. apicalis* complex is expected to reach nine cryptic species, and a complete survey over the whole distribution range of the complex might uncover many more species. Additional research on the biogeography, ecology and behaviour of these ants could also reveal species idiosyncrasies, and help us to better understand the speciation process within this Neotropical species complex. For example, one might uncover the selective forces that have driven this high diversification in the *P. apicalis* clade and not in the *P. verenae,* or elucidate if this group diverged in sympatry or if their actual distribution is derived. Finally, as evidenced in skipper butterflies [15], fig-pollinating wasps [81], cerambycid beetles [14], pseudoscorpions [13] and ants [51]–[53], our results add support to the hypothesis of a higher incidence of cryptic species in the tropics. They thus highlight the importance of large-scale studies and the necessity of testing new complementary conclusive tools to correctly quantify Neotropical biological diversity. Such research endeavours are certainly overwhelming, but they are essential for the understanding of the world's true diversity of life and the first step to assure its conservation.

## Supporting Information

Figure S1 Worker specimen of *Pachycondyla verenae* (PVE) Morph 1 from Petit Saut, French Guiana. Lateral view (A), Full-face view (B) and petiole, oblique lateral view (C).
(TIF)

Figure S2 Worker specimen of *Pachycondyla apicalis* (PAP) Morph 3 from Los Tuxlas, Mexico. Lateral view (A), Full-face view (B) and petiole, oblique lateral view (C).
(TIF)

Figure S3 Worker specimen of *Pachycondyla apicalis* (PAP) Morph 4 from Petit Saut, French Guiana. Lateral view (A), Full-face view (B) and petiole, oblique lateral view (C).
(TIF)

Figure S4 Worker specimen of *Pachycondyla apicalis* (PAP) Morph 5 from Petit Saut, French Guiana. Lateral view (A), Full-face view (B) and petiole, oblique lateral view (C).
(TIF)

Figure S5 Worker specimen of *Pachycondyla apicalis* (PAP) Morph 6 from Petit Saut, French Guiana. Lateral view (A), Full-face view (B) and petiole, oblique lateral view (C).
(TIF)

Figure S6 **Scanning electron micrographs of the stridulatory file of five morphs from the** ***Pachycondyla*** **a** ***picalis*** **species complex.** PVE Morph 1 (A), PAP Morph 3 (B), PAP Morph 4 (C), PAP Morph 5 (D) and PAP Morph 6 (E). PVE Morph 1 and PAP Morphs 4, 5, and 6 from Petit Saut, French Guiana and PAP Morph 3 from Los Tuxlas, Mexico. PVE: *P. verenae*, PAP: *P. apicalis*.
(TIF)

Audio S1 Series of chirps from a *Pachycondyla verenae* (PVE) Morph 1 worker.
(WAV)

Audio S2 Series of chirps from a *Pachycondyla apicalis* (PAP) Morph 3 worker.
(WAV)

Audio S3 Series of chirps from a *Pachycondyla apicalis* (PAP) Morph 4 worker.
(WAV)

Audio S4 Series of chirps from a *Pachycondyla apicalis* (PAP) Morph 5 worker.
(WAV)

Audio S5 Series of chirps from a *Pachycondyla apicalis* (PAP) Morph 6 worker.
(WAV)
