# Supplementary figures and images for: Stridulations Reveal Cryptic Speciation in Neotropical Sympatric Ants
Source: PLoS One. 2010 Dec 22;5(12):e15363. doi: 10.1371/journal.pone.0015363 (PMC3008743; doi:10.1371/journal.pone.0015363)

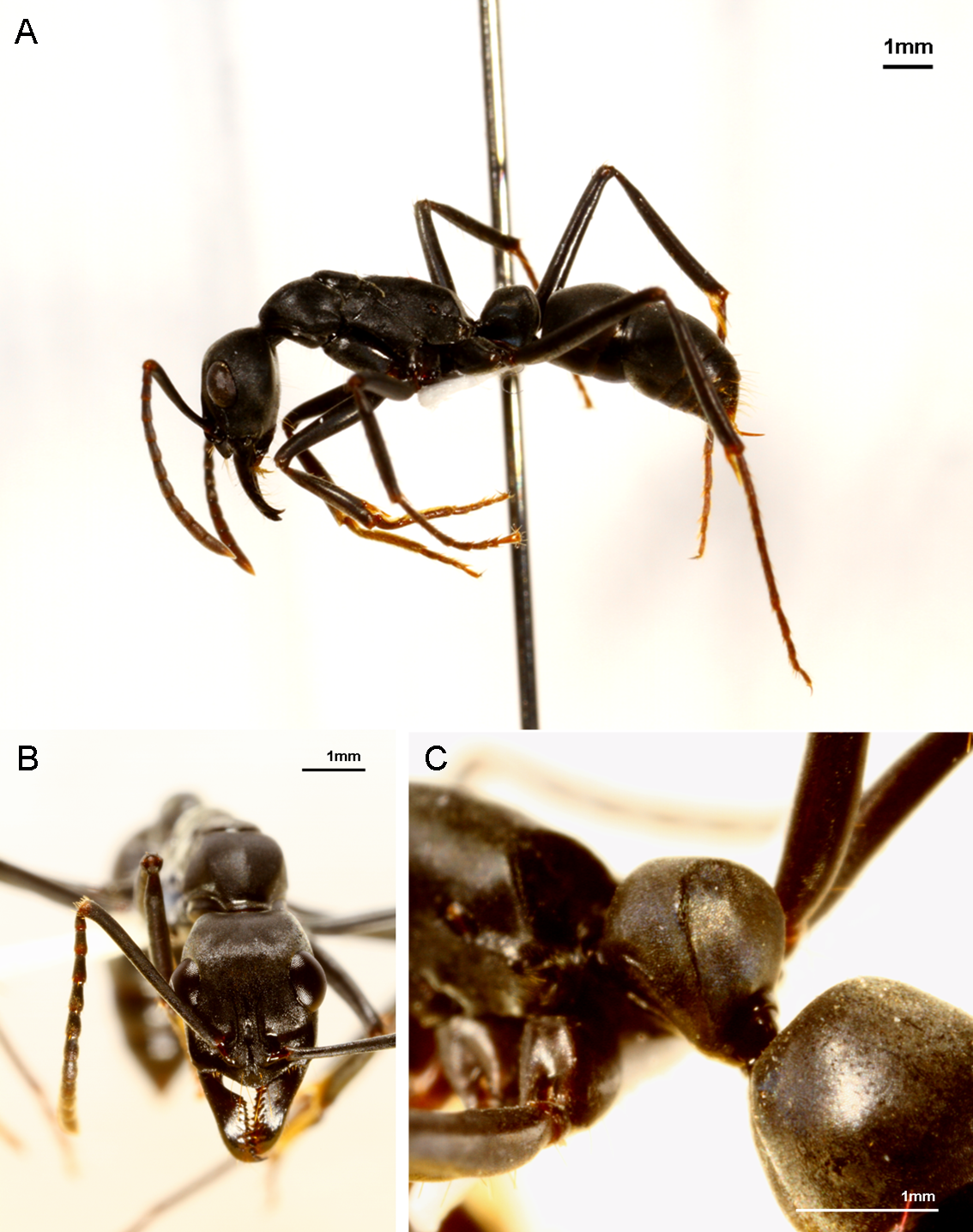

Supplement: Figure S1 — Worker specimen of Pachycondyla verenae (PVE) Morph 1 from Petit Saut, French Guiana. Lateral view (A), Full-face view (B) and petiole, oblique lateral view (C). (TIF) [file pone.0015363.s001.tif]

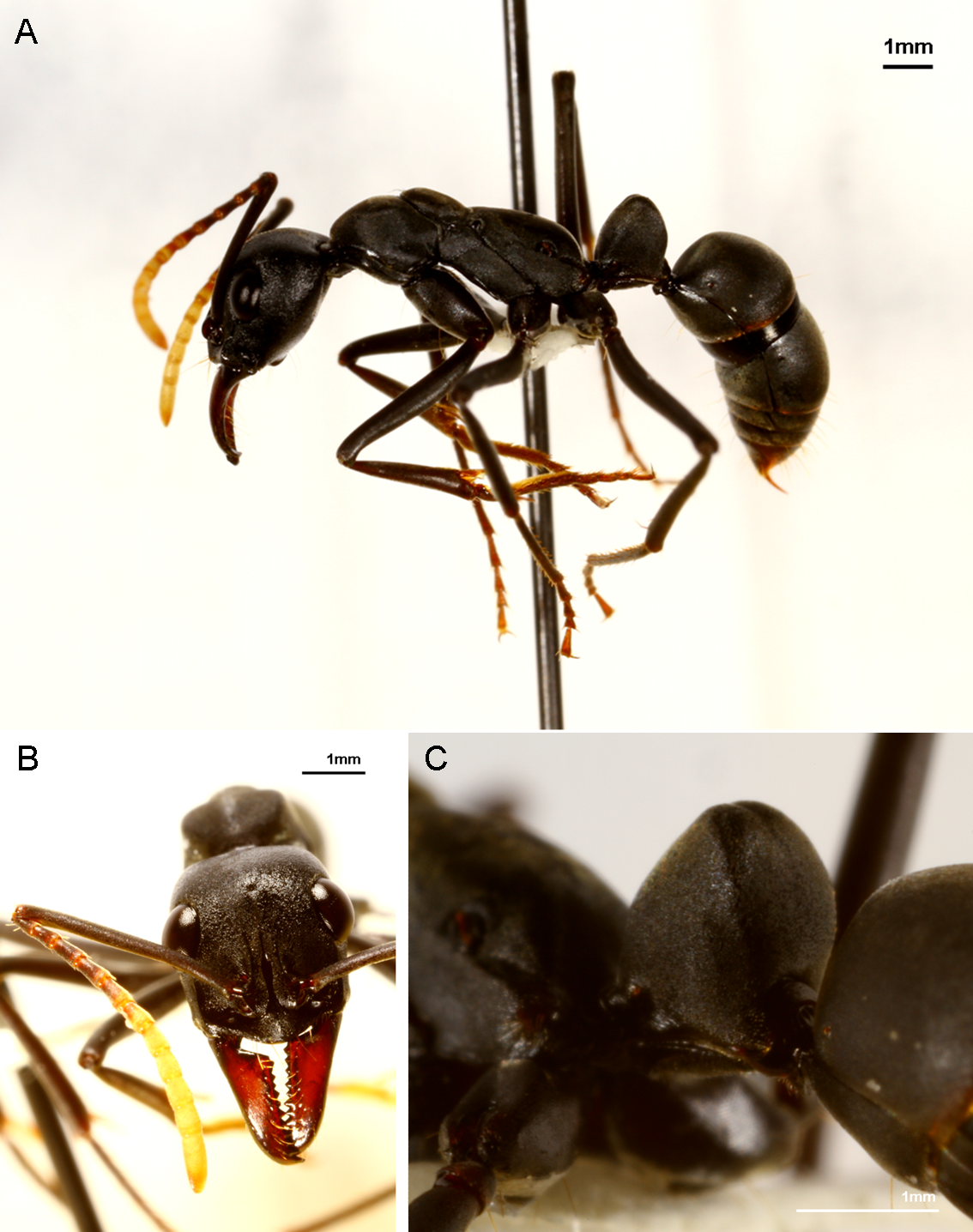

Supplement: Figure S2 — Worker specimen of Pachycondyla apicalis (PAP) Morph 3 from Los Tuxlas, Mexico. Lateral view (A), Full-face view (B) and petiole, oblique lateral view (C). (TIF) [file pone.0015363.s002.tif]

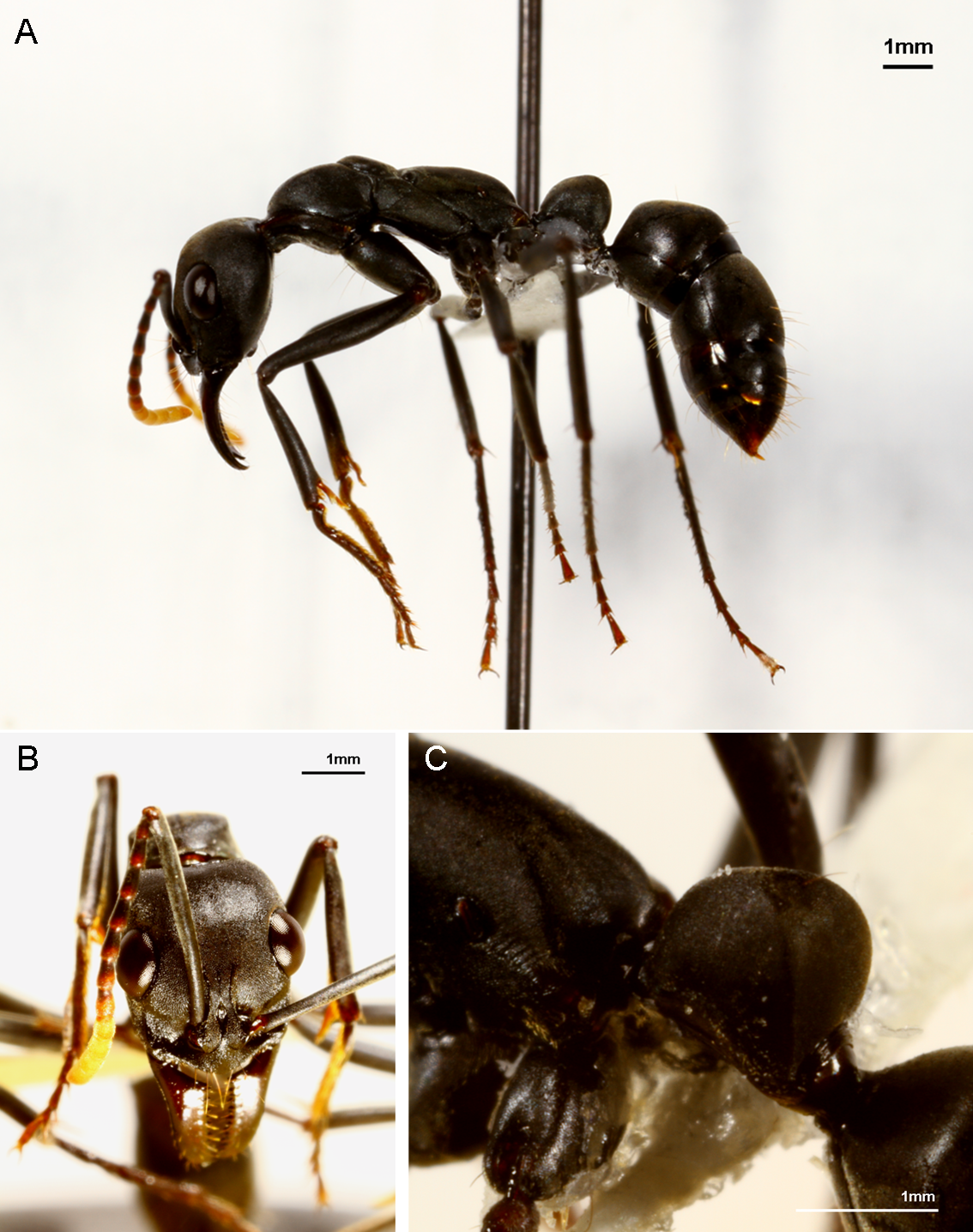

Supplement: Figure S3 — Worker specimen of Pachycondyla apicalis (PAP) Morph 4 from Petit Saut, French Guiana. Lateral view (A), Full-face view (B) and petiole, oblique lateral view (C). (TIF) [file pone.0015363.s003.tif]

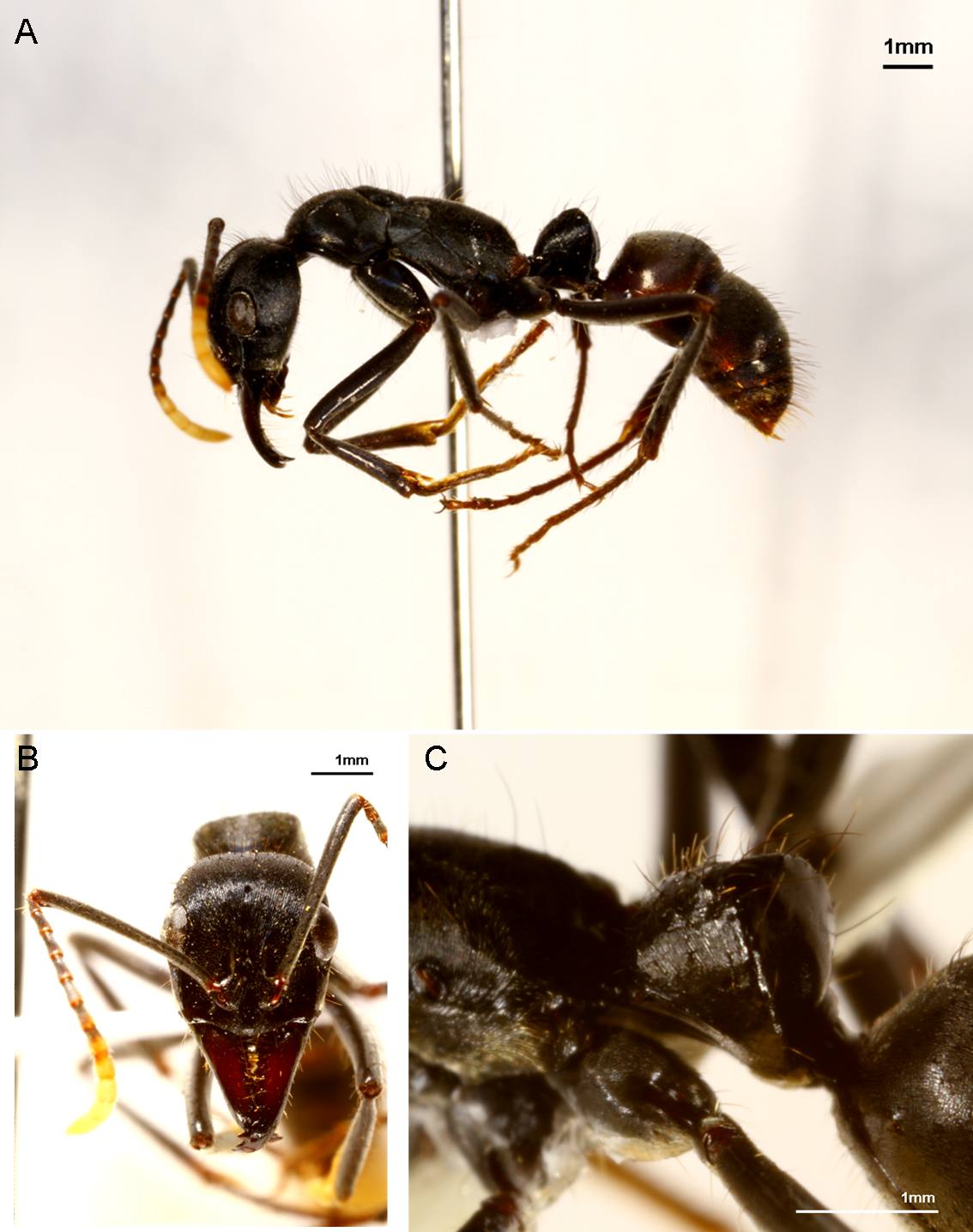

Supplement: Figure S4 — Worker specimen of Pachycondyla apicalis (PAP) Morph 5 from Petit Saut, French Guiana. Lateral view (A), Full-face view (B) and petiole, oblique lateral view (C). (TIF) [file pone.0015363.s004.tif]

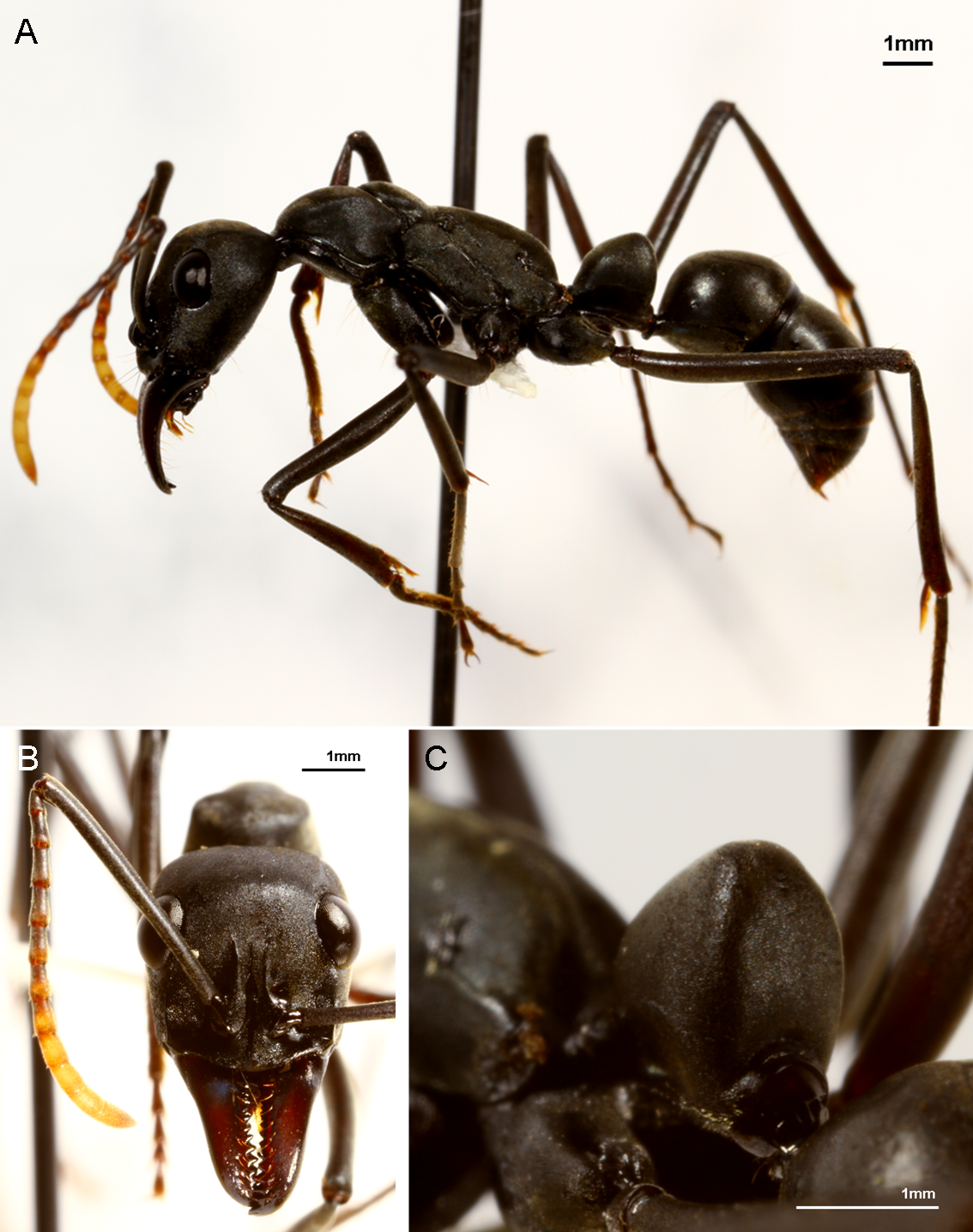

Supplement: Figure S5 — Worker specimen of Pachycondyla apicalis (PAP) Morph 6 from Petit Saut, French Guiana. Lateral view (A), Full-face view (B) and petiole, oblique lateral view (C). (TIF) [file pone.0015363.s005.tif]

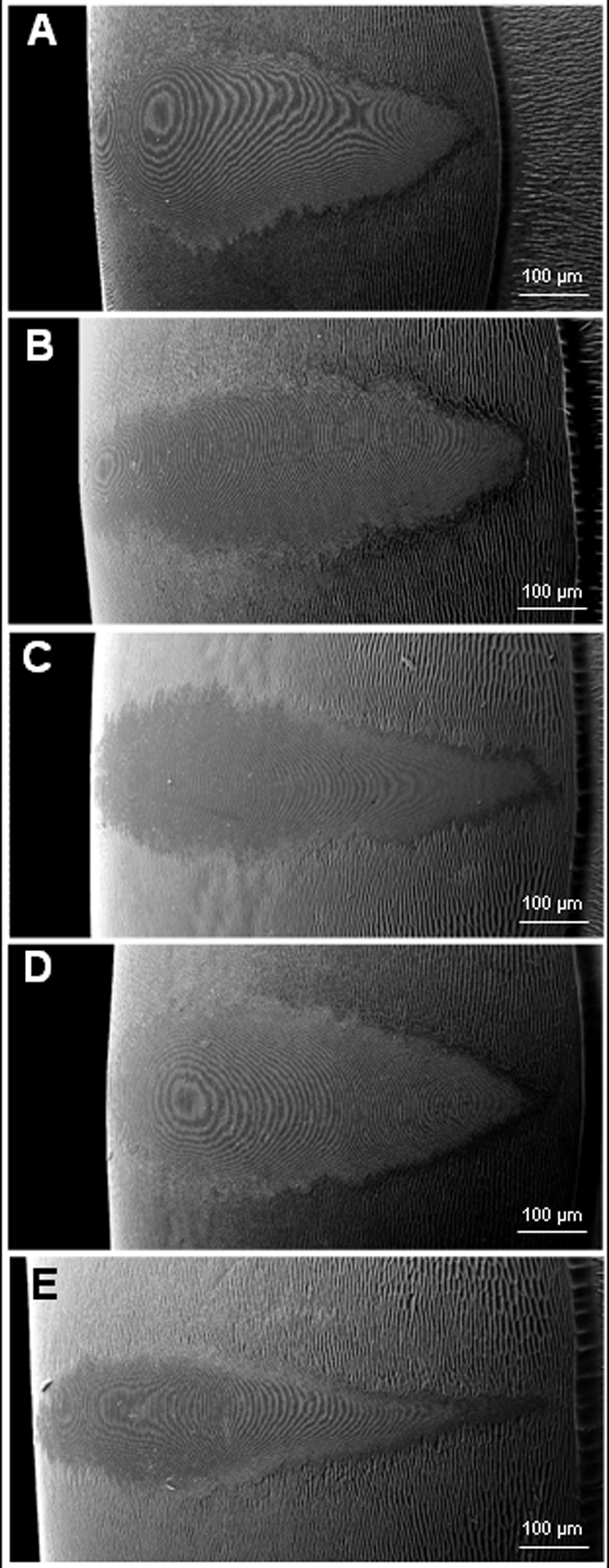

Supplement: Figure S6 — Scanning electron micrographs of the stridulatory file of five morphs from the Pachycondyla a picalis species complex. PVE Morph 1 (A), PAP Morph 3 (B), PAP Morph 4 (C), PAP Morph 5 (D) and PAP Morph 6 (E). PVE Morph 1 and PAP Morphs 4, 5, and 6 from Petit Saut, French Guiana and PAP Morph 3 from Los Tuxlas, Mexico. PVE: P. verenae, PAP: P. apicalis. (TIF) [file pone.0015363.s006.tif]
